# Predictive value of gastrointestinal symptoms and patient risk factors for NSAID-associated gastrointestinal ulcers defined by endoscopy? Insights from a pooled analysis of two naproxen clinical trials

**DOI:** 10.1371/journal.pone.0284358

**Published:** 2023-04-13

**Authors:** Mart A. F. J. van de Laar, Rainer Schöfl, Marlou Prevoo, Jan Jastorff

**Affiliations:** 1 Department of Psychology, Health and Technology, University of Twente, Enschede, The Netherlands; 2 Department of Internal Medicine IV, Ordensklinikum Barmherzige Schwestern, Linz, Austria; 3 Grünenthal GmbH, Aachen, Germany; Hospital Guillermo Kaelin de la Fuente, PERU

## Abstract

**Objective:**

Nonsteroidal anti-inflammatory drugs (NSAIDs) are widely used to treat pain and rheumatic conditions. To facilitate patient management, we determined the predictive value of gastrointestinal (GI) symptoms and risk factors for the development of NSAID-associated GI injuries.

**Methods:**

Post-hoc analysis of pooled data from naproxen treatment arms of two identical, randomized, double-blind, controlled phase 3 trials in arthritis patients at risk of GI adverse events. Endoscopic incidence of GI ulcers at baseline, and 1, 3, and 6 months was employed as a surrogate parameter for GI injury. For GI symptom analysis, Severity of Dyspepsia Assessment questionnaire was used. For GI risk factor analysis, the high risk factors: previous GI injury, concomitant selective serotonin reuptake inhibitors or corticosteroids, ulcer history, concomitant low-dose aspirin, and age >65 years were employed.

**Results:**

Data of 426 naproxen patients were analyzed. Distribution of GI symptoms between patients with and without ulcer was similar; about one third of patients developing an ulcer reported no GI pain symptoms. GI symptoms experienced under naproxen treatment were thus not indicative of GI injury. The proportion of patients developing an ulcer increased with the number of risk factors present, however, about a quarter of patients without any of the analyzed risk factors still developed an ulcer.

**Conclusion:**

GI symptoms and the number of risk factors are not reliable predictors of NSAID-induced GI injury to decide which patients need gastroprotection and will lead to a large group of patients with GI injuries. A preventive rather than reactive approach should be taken.

## Introduction

Nonsteroidal anti-inflammatory drugs (NSAIDs) are among the most commonly used medications worldwide [1, 2]. Millions of people use prescription or over the counter (OTC) NSAIDs every day for their analgesic, anti-inflammatory and anti-pyretic properties in a wide range of conditions [3, 4]. In particular, both non-selective and Cox-2-selective NSAIDs are frequently used for the management of pain and inflammation in chronic arthritic conditions such as rheumatoid arthritis and osteoarthritis [5]. Although very effective, NSAIDs can, however, be associated with gastrointestinal (GI) complications such as GI ulcer development, which primarily affect the stomach and upper intestine. Their use may lead to significant morbidity as a result of bleeding, perforation, and obstruction, and even potentially death [3, 6, 7]. Incidence rates are high, with 15%-30% of long-term NSAID users developing endoscopic ulcers, and clinically relevant complications occurring in 2%-4% of ulcers [8]. NSAIDs increase the risk of upper gastrointestinal complications by 2–4 times, with COX-2 inhibitors yielding a lower risk compared to non-selective NSAIDs [9, 10]. The risk further increases if combined with low-dose aspirin, corticosteroids or selective serotonin reuptake inhibitors (SSRIs) [9, 10] and is also influenced by the dose of the NSAID [10–12]. Mortality rates following upper GI complications associated with the use of NSAIDs have been estimated between 5% and 21% [3, 6, 9].

In order to mitigate NSAID-associated GI side-effects, most guidelines and recommendations for pain treatment advise on the concomitant administration of proton pump inhibitors (PPIs) for gastroprotection, mainly for at risk patient groups (see [13] for overview; [14–17]). Gastroprotective strategies have been shown to be effective in reducing the risk of NSAID-associated GI side-effects [18, 19], however, two major challenges to reduce serious GI complications in at-risk patients are the low prescription rates of preventive therapy and poor patient adherence to prescribed gastroprotective agents. Despite existing recommendations, about 50% of NSAID patients are not prescribed adequate or even any gastroprotection [20]. Moreover, even if gastroprotective medication is prescribed, 15–30% of patients are not compliant, with main reasons being forgetfulness and low GI pain intensity [21, 22].

GI injury following the use of NSAIDs may be preceded by symptoms such as gastroesophageal reflux, belching, bloating, nausea and epigastric discomfort, however, a large percentage of patients do not experience symptoms and yet develop GI complications. More than 50% of NSAID users with serious peptic ulcer complications had no previous warning symptoms, whereas many patients with gastric symptoms may not in fact suffer from any mucosal damage [23–25]. GI injury and thereby the need for gastroprotection can thus not be predicted on the basis of symptoms experienced by the patients. It was therefore suggested that the presence of risk factors could determine if patients require preventive gastroprotective measures with NSAID use [3, 26]. Risk factors for the development of GI complications under NSAID medication include age >65 years, high-dose or multiple NSAID use, history of ulcers, serious comorbidity (e.g., cardiovascular disease, hepatic or renal impairment, diabetes, hypertension), duration of NSAID use, concomitant use of certain medications (low-dose aspirin, corticosteroids, anticoagulants, SSRIs) and smoking, excessive alcohol consumption and Helicobacter pylori infection [4, 7, 27, 28].

To support healthcare professionals in the identification of patients in need of gastroprotection and to verify the relation between gastric symptoms and ulcer development, we performed a post-hoc analysis, pooling data from two clinical trials comparing GI safety between a fixed dose combination of naproxen and esomeprazole and enteric coated (EC) naproxen alone in patients at increased GI risk. Both trials showed that the cumulative observed incidence of gastric ulcers over 6 months was significantly lower in patients treated with the fixed dose combination compared with those treated with EC naproxen, with a relative risk reduction of 82.3% and 70.8%, respectively [29]. Focusing on the EC naproxen arm of these two trials in this post hoc analysis, allowed us to investigate the value of individual risk factors for predicting the development of gastric ulcers defined by endoscopy, and the distribution of gastric symptoms reported by the patients depending on whether they developed gastric ulcers or not.

## Patients and methods

### Data selection

Data were extracted from the data sets of two identical randomized, double-blind, parallel-group, controlled, multicenter, phase 3 trials (NCT00527787 and NCT01129011) conducted in accordance with the Declaration of Helsinki. Trial protocols and amendments were reviewed and approved by the New England Institutional Review Board in Wellesley, MA. Participating patients provided written informed consent prior to enrolment. The trials evaluated the incidence of gastric and duodenal ulcers under treatment with a fixed-dose combination of EC naproxen and immediate-release esomeprazole magnesium compared to EC naproxen alone in patients at risk of developing NSAID-associated ulcers [29]. Helicobacter pylori-negative (stool antigen test) patients aged ≥50 years or 18–49 years with a history of uncomplicated gastric or duodenal ulcer within the past 5 years, who had osteoarthritis, rheumatoid arthritis, or another condition expected to require daily NSAID treatment for at least 6 months were eligible to participate. Screening endoscopy showing any gastric or duodenal ulcer at least 3 mm in diameter with depth led to exclusion. Further exclusion criteria have been published elsewhere [29]. Eligible patients received either the fixed-dose combination 500 mg naproxen/20 mg esomeprazole or naproxen 500 mg twice daily for 6 months or until a gastrointestinal ulcer was detected. In the latter case, the patient discontinued the trial and was considered a trial completer.

### Assessments

The present post-hoc analysis used the endoscopic incidence of gastric and duodenal ulcers as parameter for GI injury at the predefined timepoints baseline, and 1, 3, and 6 months after start of trial medication. In case the participant left the trial prematurely, an additional endoscopy was performed. An ulcer was defined as a mucosal break of at least 3 mm in diameter (measured by close application of open endoscopic biopsy forceps) with unequivocal crater depth [30].

For the analysis of GI symptoms, original trial data from the following assessments were included: patient-reported outcome questionnaire Severity of Dyspepsia Assessment (SODA) at baseline, 1, 3, and 6 months, and the occurrence of predefined NSAID-associated upper GI (UGI) adverse events (AEs). The SODA questionnaire is a self-administered multidimensional measure of dyspepsia-related health that was designed as a primary outcome measure for randomized clinical trials [31, 32]. It consists of the 3 domains pain intensity, non-pain symptoms, and satisfaction. The reliability, validity and responsiveness of SODA as a measure of dyspepsia has been demonstrated, with the pain intensity domain showing excellent reliability (α = 0.93), fair to good reproducibility (interclass correlation coefficient = 0.49) and the highest responsiveness (AUC = 0.78) out of the three SODA domains [32]. Data regarding the first 2 domains were analyzed here: pain intensity (6 questions about abdominal discomfort; score 2–47) and non-pain symptoms (7 questions about burping/belching, heartburn, bloating, passing gas, sour taste, nausea, bad breath; score 7–35). Predefined NSAID-associated UGI AEs are listed in S1 Appendix.

For the GI risk factor analysis, the following five high risk factors were taken into account:

- Previous GI injury: the preferred terms from the medical history recorded in the case report forms (CRFs) were duodenitis, erosive duodenitis, gastric hemorrhage, gastric ulcer, perforation, gastritis erosive, gastritis hemorrhagic, GI erosion, GI hemorrhage, hematemesis, melena, and UGI hemorrhage.
- Concomitant medication: selective serotonin reuptake inhibitors (SSRIs; ACT code N06AB) or corticosteroids (ATC code H02).
- Ulcer history as recorded in the CRF.
- Low-dose aspirin comedication as recorded in the CRF.
- Age >65 years.

### Statistical analysis

All statistical analyses were performed using SAS version 9.4 (SAS Institute Inc., Cary, NC). Analyses were conducted only with data of the naproxen arm of the original trials. The analyzed safety population consisted of all randomized patients who received at least one dose of naproxen.

Endoscopic incidence of gastric and duodenal ulcers was used as parameter for GI injury. As the proportion of duodenal ulcers was very small, results for gastric and duodenal ulcers were combined.

SODA pain intensity scores are presented as scatterplots stratified by ulcer development for the timepoints baseline, 1 month, and 3 months. The timepoint final visit (6 months) also includes patients who discontinued prematurely for any reason but who had developed an ulcer at that time. P values for the difference between patients with and without ulcer development were calculated using two-sided Wilcoxon rank sum tests.

For the GI risk factor analysis, calculations of relative risk with 95% confidence intervals (CIs) were carried out. A binomial multiple logistic regression was performed showing the relative risks with 95% CIs and two-sided P values for all factors. Differences were considered significant with a P <0.05.

## Results

The two original trials included 426 naproxen patients; the majority (82.6%) received the trial medication for the treatment of osteoarthritis pain (Table 1). A quarter of the patients (108 patients [25.4%]) were >65 years old, 37 (8.7%) had a history of ulcers, 133 (31.2%) had previous GI injuries, 60 (14.1%) received SSRIs or corticosteroids, and 102 (23.9%) took low-dose aspirin. Of the 426 naproxen patients, 119 patients (27.9%) developed an ulcer during the trial.

**Table 1:** Patient demographics and baseline characteristics of the two trial populations receiving enteric-coated naproxen (pooled data; safety set).

	Naproxen (n = 426)
Sex	
Female	291 (68.3%)
Male	135 (31.7%)
Age (years)	60.6±8.4
Body mass index (kg/m^2^)	30.9±6.1
Smoker	65 (15.3%)
Low-dose aspirin use	102 (23.9%)
Indication for NSAID use^a^	
Osteoarthritis	352 (82.6%)
Rheumatoid arthritis	17 (4%)
Ankylosing spondylitis	2 (0.5%)
Other	95 (22.3%)
Documented history of ulcer	
Gastric	31 (7.3%)
Duodenal	5 (1.2%)
Both	1 (0.2%)

Data are mean ± standard deviation or number of patients (%).

NSAID, nonsteroidal anti-inflammatory drug

^a^ Patients may have had more than one indication for NSAID use.

### GI symptoms

As patients developing an ulcer discontinued the trial, the analysis of GI symptoms was split in two parts. Analysis at visits baseline, 1 month and 3 months provides insights into whether symptoms are indicative of ulcer development at a later timepoint. Analysis of the final visit provides insights into whether symptoms are indicative of an ulcer present at that same point in time.

**Fig 1:**
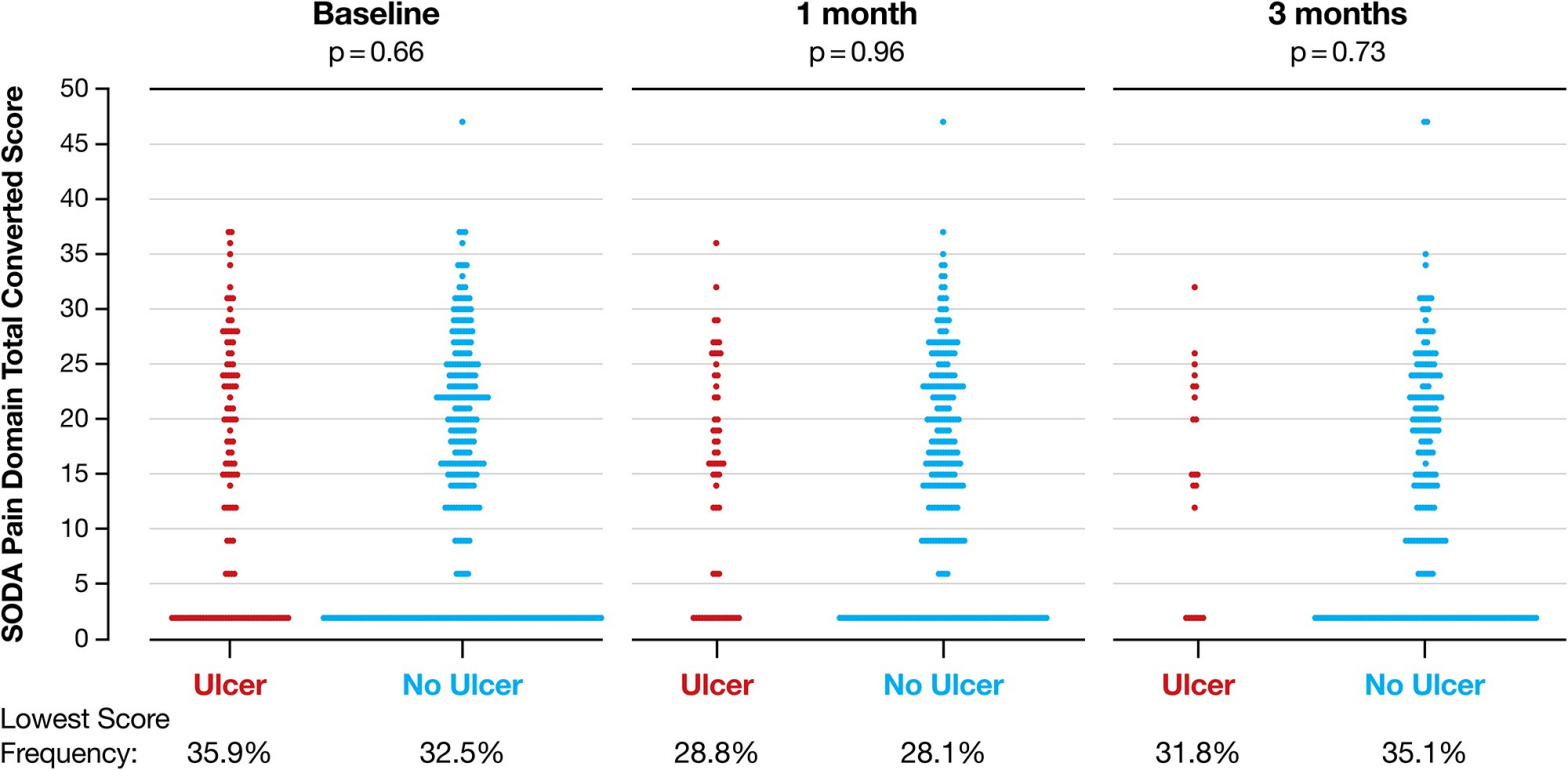
SODA pain intensity scores preceding ulcer development stratified by ulcer development at baseline, 1 month, and 3 months of treatment with naproxen. Data are number of patients. SODA, Severity of Dyspepsia Assessment.

Fig 1 shows the SODA pain intensity scores preceding ulcer development at baseline, 1 month, and 3 months. At each timepoint, about a third of the patients in both groups (patients with ulcers, patients without ulcers) did not suffer from gastrointestinal pain (score of 2 out of 47): there were no significant differences at any timepoint between the number of patients developing ulcers (baseline 35.9%, 1 month 28.8%, 3 months 31.8%) and patients without ulcers (baseline 32.5%, 1 month 28.1%, 3 months 35.1%; all p>0.65). Thus, pain symptoms were not reliable in predicting the development of GI injury.

**Fig 2:**
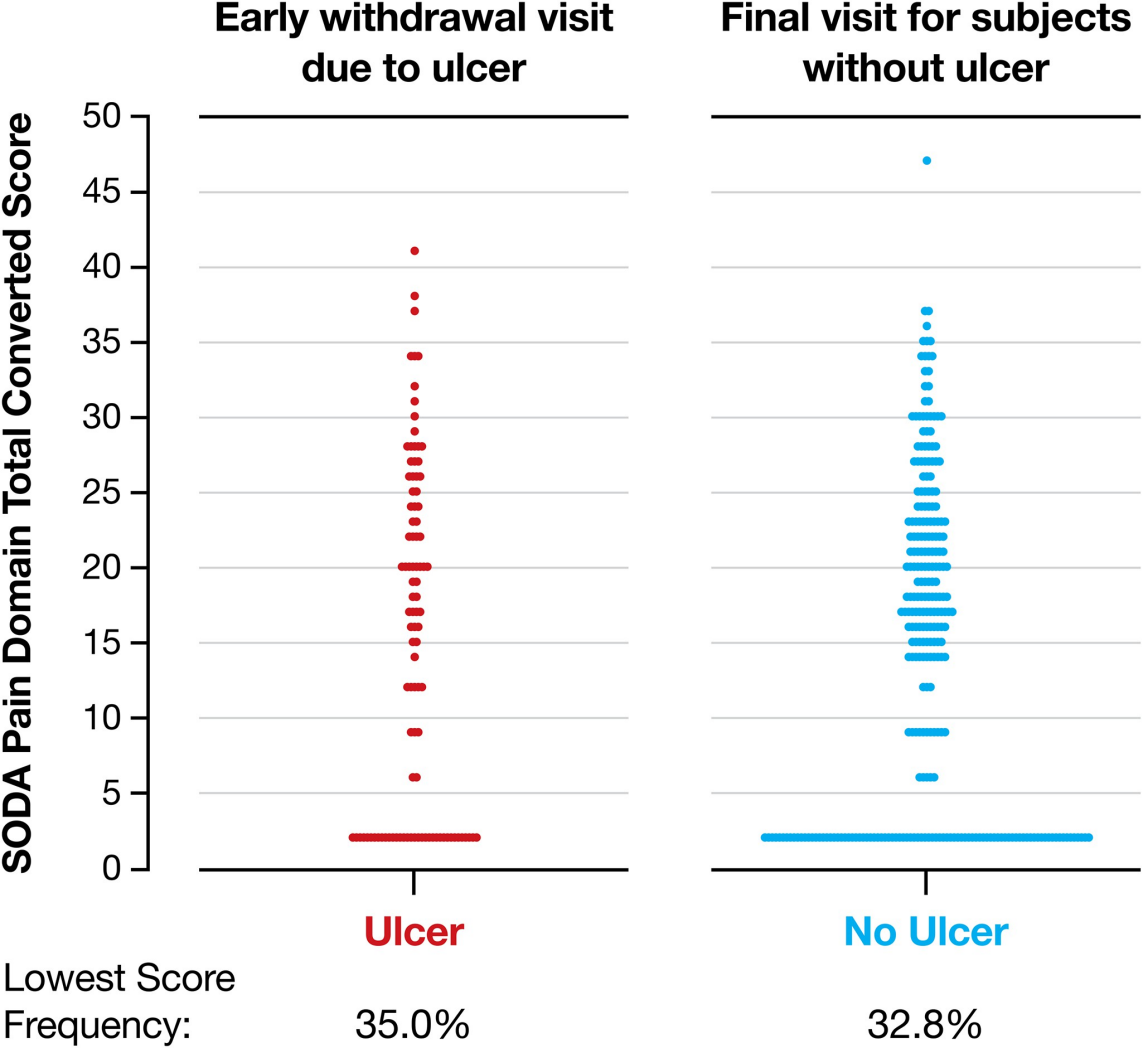
SODA pain intensity scores of patients who had an ulcer either at the final visit of the study (6 months) or at premature study exit for any other reason or completed the study without ulcer. Data are number of patients. SODA, Severity of Dyspepsia Assessment.

There were also no differences in SODA pain symptoms between the groups at the final visit (Fig 2). The ulcer group comprises patients who either had an ulcer at one of the visits or discontinued prematurely for any other reason but with an ulcer present at that final visit. Patients in the no ulcer group completed the trial without an ulcer. The comparison shows that symptoms were also not indicative of ulcer presence at the moment of detection of the ulcer (32.8% vs 35.0% for no ulcer and ulcer groups, respectively).

An analogous analysis, focusing on the ‘non-pain symptoms’ of the SODA score yielded similar results. There were no differences between the groups at any timepoint, with between 12% to 15% of the patients reporting no non-pain symptoms (all p>0.40; results not shown).

The proportion of patients with NSAID-associated UGI AEs under naproxen also did not differ notably between patients developing ulcers and patients without ulcers (Table 2). Prespecified GI related AEs did not occur in approximately one third of patients in both groups (patients with ulcer 34.5%, patients without ulcer 32.9%).

**Table 2:** Proportion of patients with NSAID-associated upper gastrointestinal adverse events under naproxen treatment (n = 426).

GI-related adverse events	Patients without ulcer (n = 307)	Patients with ulcer (n = 119)
None	101 (32.9%)	41 (34.5%)
1	72 (23.5%)	38 (31.9%)
2	64 (20.8%)	27 (22.7%)
3	41 (13.4%)	7 (5.9%)
4	15 (4.9%)	5 (4.2%)
5	9 (2.9%)	1 (0.8%)
6	2 (0.7%)	0
7	3 (1%)	0

Data are number of patients (%). GI, gastrointestinal; NSAID, nonsteroidal anti-inflammatory drug

Overall, these analyses indicate that GI symptoms are not a reliable indicator for GI injury. The distribution of symptoms between the patients with and without ulcer was similar. More importantly, about one third of patients developing an ulcer reported no GI pain symptoms.

### GI risk factor analysis

The proportion of patients developing an ulcer compared to the ones not developing an ulcer was similar for the risk factors previous GI injury, concomitant medication, low-dose aspirin, and age >65 years (Table 3). Thus, none of these risk factors was a predictor for ulcer development, as indicated by the non-significant relative risk. The only risk factor predictive of ulcer development in the studies was the presence of ulcer history, increasing the risk by a factor of 2.

**Table 3:** Ulcer development under naproxen treatment (n = 426).

Risk factor	Ulcer development without risk factor	Ulcer development with risk factor	Mean estimated relative risk^a^
Previous GI injury	77/293 (26.3%)	42/133 (31.6%)	1.2 (0.88, 1.65)
Concomitant medication	107/366 (29.2%)	12/60 (20%)	0.68 (0.4, 1.16)
Ulcer history	100/389 (25.7%)	19/37 (51.4%)	2.0 (1.4, 2.85)
Low-dose aspirin	86/324 (26.5%)	33/102 (32.4%)	1.22 (0.87, 1.7)
Age >65 years	85/318 (26.7%)	34/108 (31.5%)	1.18 (0.84, 1.64)

Data are number of patients (%) and mean estimated relative risk (95% CIs).

^a^ risk of exposed vs. unexposed to risk factor; CI, confidence interval; GI, gastrointestinal

Results were similar, when all risk factors were included in one model, correcting for each other. Again, the only risk factor predictive of ulcer development was the presence of ulcer history (Table 4). To determine the influence of age, we evaluated an additional model, defining age not as a categorical, but as a continuous variable (Table 4). In this case, the mean estimated relative risk of 1.02 (95%CI 1.00, 1.04) was statistically significant (p = 0.0171), indicating that with increasing age, the risk of ulcer development of the patient increases. It should, however, be noted that the lower 95% CI touches 1.00.

**Table 4:** Relative risk models including all five high risk factors.

Risk factor	Mean estimate	95% CI	P value
**Model with age >65 years**			
Previous GI injury	1.14	0.83, 1.55	0.42
Concomitant medication	0.67	0.40, 1.14	0.14
Ulcer history	1.97	1.37, 2.82	0.0002
Low-dose aspirin	1.31	0.95, 1.81	0.10
Age >65 years	1.20	0.87, 1.66	0.26
**Model with age as a continuous variable**			
Previous GI injury	1.15	0.84, 1.56	0.38
Concomitant medication	0.67	0.39, 1.13	0.13
Ulcer history	2.09	1.47, 2.97	<0.0001
Low-dose aspirin	1.29	0.93, 1.79	0.12
Age continuous	1.02	1.00, 1.04	0.02

CI, confidence interval; GI, gastrointestinal

With individual risk factors providing limited predictive value for ulcer development, we further investigated, whether the number of risk factors present in each patient would be more informative. Of the 307 patients who did not develop an ulcer, 34.2% did not present with any of the five risk factors, and 41%, 18.6%, 5.2%, and 1% had one, two, three or four risk factors, respectively (Table 5). Thus, the majority of patients (75.2%) who did not develop an ulcer had zero or only one risk factor. Importantly, this result was similar for the 119 patients who developed an ulcer during the trial: Also in this group, the majority of patients (65.6%) presented with zero or only one risk factor (Table 5). Frequencies were also comparable between the groups when more than one risk factor was present. Nevertheless, with increasing number of risk factors, the risk of developing an ulcer increased from 27.2% for patients with one risk factor to 40.7% for patients presenting with 3 risk factors. However, the increasingly lower sample sizes for patients with 2 or more risk factors need to be considered. More importantly, our analysis also showed that out of all patients without any of the analyzed risk factors present (n = 136), 22.8% developed an ulcer.

**Table 5:** Influence of the number of risk factors on ulcer development (n = 426).

Number of risk factors	0	1	2	3	4
No ulcer development	105	126	57	16	3
(n = 307)	(34.2%)	(41%)	(18.6%)	(5.2%)	(1%)
Ulcer development	31	47	30	11	0
(n = 119)	(26.1%)	(39.5%)	(25.2%)	(9.2%)	-
Ulcer development with number of risk factor	31/136	47/173	30/87	11/27	0/3
	(22.8%)	(27.2%)	(34.5%)	(40.7%)	-

Data are number of patients (%). Zero risk factor denotes that patients were younger than 65 years.

## Discussion

Our data show no relationship between the presence of GI symptoms (pain and non-pain symptoms measured by SODA and NSAID-associated UGI AEs) and the development of peptic ulcers and thereby add to the body of evidence that GI symptoms are not a reliable indicator for upper GI injury [23–25]. For example, Sostres and colleagues showed that NSAID use increased the risk of upper GI bleeding events by 4.9 and that events were not preceded by dyspeptic warning symptoms in over 60% of patients [25]. The majority of patients in that study had been short-time NSAID users with a median duration of NSAID use of 15 days (mostly OTC for reasons other than chronic rheumatic disease). The observation that short-term NSAID treatment also carries a high risk of GI adverse events is supported by the phase 3 trials underlying the present analysis. Forty-one percent of the gastrointestinal ulcers were present already at the first visit, after one month of treatment [29]. Similarly, Lewis et al. reported that the risk of gastrointestinal bleeding is highest during the first week of treatment and decreases thereafter [33]. These observations agree with our experience in clinical practice. Patients who develop symptomatic ulcers or complications are more likely to discontinue their NSAID medication. On the other hand, due to selection, long-term users of NSAIDs are more likely patients who tolerate the medication. Increasing awareness on the patients’ side that gastrointestinal complications are often not preceded by symptoms may improve compliance to gastroprotection, which is often suboptimal [21, 22].

Our analysis also confirmed that the risk of ulcer development increases with the number of the risk factors present; however, the increasingly lower sample sizes need to be considered. From the five preselected risk factors, ulcer history and increasing age when modelled continuously were predictive of ulcer development. Strict age limitations such as >65 years are not supported by our data. Most importantly however, about a quarter of the patients without a risk factor present developed an ulcer. Thus, even younger patients without any of the five preselected high-risk factors for NSAID-associated GI ulcers investigated in our analysis may still develop GI complications under high dose NSAID therapy. Our data confirm that despite risk factors, any prediction model lacks clinical relevance, since all patients receiving high dose naproxen were at risk of developing GI injury. Thus, we found no indication that only patients presenting with risk factors should receive gastroprotection.

While our analysis focused on naproxen, all non-selective NSAIDs increase the risk of gastrointestinal adverse events. Masclee et al. reported that treatment with non-selective NSAIDs was associated with an increased relative risk of 4.3 compared to no NSAID use, based on a case series analysis of data from 114,835 patients with upper gastrointestinal bleeding [10]. Bhala and colleagues performed a meta-analysis of randomized controlled trials, including more than 350.000 patients [9]. They showed that all COX-2 selective and non-selective NSAIDs significantly increased the risk of upper gastrointestinal complications, with a rate ratio of 1.81 for coxibs, 1.89 for diclofenac, 3.97 for ibuprofen and 4.22 for naproxen respectively [9]. Thus, the low reliability of GI risk factors in predicting which patient will develop GI adverse events is likely applicable also to other non-selective NSAIDs.

Co-prescription of PPIs with non-selective NSAIDs is recommended by national and international guidelines to minimize the risk of ulcer development [13–17, 34]. There are, however, differences regarding recommended patient groups, and most guidelines focus recommendations on patients at risk of developing GI injury. Despite these recommendations, adherence to PPI co-prescription is rather low with recent studies from the UK and Belgium indicating that even more than 50% of patients with a history of GI disease or at high risk of gastrointestinal adverse events do not receive gastroprotection [35, 36].

Although co-prescription of PPIs for gastroprotection appears to have improved since the start of the century from under 8% [37] to 44–49% [20, 38, 39], there is still room for improvement. Patient and physician education are still required and a fixed dose combination of NSAIDs plus a PPI might be a way forward to ascertain adherence to prescription guidelines and patient compliance [20]. The fixed-dose combination of EC naproxen and esomeprazole has been shown to provide effective pain relief in patients with osteoarthritis [40, 41]. It also significantly reduced the incidence of dyspepsia and gastric or gastroduodenal ulcers, regardless of low-dose aspirin use, compared to naproxen and other NSAIDs [29, 40–45] with an established long-term safety profile [46].

Our study results have to be interpreted with care because of its nature as a post-hoc analysis of two clinical trials with a restricted number of patients. Real world evidence studies are needed to determine if routine addition of GI protection to NSAID therapy, regardless of GI risk factors, is cost-effective.

The incidence of endoscopic gastric ulcers was used as surrogate endpoint for ulcer complications, rather than actual perforations, obstructions, or bleedings. The clinical relevance of endoscopic ulcers has been debated, mainly because studies showing direct progression from endoscopic ulcers to ulcer complications are lacking [47]. Nevertheless, a multitude of studies provide links between endoscopic ulcers and more serious upper gastrointestinal harm within the context of the use of aspirin or NSAIDs [48, 49].

The original studies were conducted under slightly artificial conditions because H. pylori infection as an independent risk factor for gastric complications was excluded, whereas approximately 20–40% of individuals in the general population of Western countries are H. pylori positive [50, 51]. This means that one potentially confounding factor was absent in our study and the risk in general clinical practice may be even higher. Moreover, long-term NSAID intake is an additional risk factor (or could also be seen as a protective factor, selecting those with good tolerance to NSAIDs) and most patients probably used NSAIDs at some time prior to study start, which was not accounted for in this analysis. However, this does not change our finding that a rather high gastrointestinal risk exists even for patients presenting with none of the five high GI risk factors investigated in this study.

## Conclusion

Our data show that GI symptoms experienced under treatment with the nonselective NSAID naproxen were not indicative of GI injury. Furthermore, the percentage of patients developing an ulcer increased with the number of risk factors present. Most notably, however, about a quarter of the patients without any of the classical risk factors developed an ulcer under high dose NSAID therapy. We therefore conclude that solely relying on the presence and the number of risk factors is not a reliable guidance to decide which patient needs gastroprotection and will lead to a large group of patients with GI injuries. A preventive rather than a reactive approach should be taken. If NSAID prescription is required in a patient, the prescribing physician better assures that the patient is using gastroprotection.

## Supporting information

S1 Appendix Predefined NSAID-associated upper gastrointestinal adverse events. (DOCX)
