# Supplementary material for: Predictive value of gastrointestinal symptoms and patient risk factors for NSAID-associated gastrointestinal ulcers defined by endoscopy? Insights from a pooled analysis of two naproxen clinical trials
Source: PLoS One. 2023 Apr 13;18(4):e0284358. doi: 10.1371/journal.pone.0284358 (PMC10101403; doi:10.1371/journal.pone.0284358)
Supplement: S1 Appendix — (DOCX) [file pone.0284358.s001.docx]

**S1 Appendix.** Predefined NSAID-associated upper gastrointestinal adverse events.

| Abdominal discomfort, abdominal pain, abdominal tenderness, duodenal hemorrhage, duodenal scarring, DU hemorrhage, duodenitis, dyspepsia, epigastric discomfort, erosive duodenitis, erosive esophagitis, erosive gastritis, esophageal discomfort, esophageal disorder, esophageal hemorrhage, esophageal stenosis, esophageal ulcer, esophageal varices, esophagitis, gastric hemorrhage, gastric mucosal lesion, gastritis, gastroesophageal reflux disease, gastroesophagitis, GI erosion, GI hemorrhage, GI mucosal disorder, hemorrhagic duodenitis, hemorrhagic gastritis, hyperchlorhydria, nausea, reflux esophagitis, stomach discomfort, upper abdominal pain, vomiting |
| --- |

DU, duodenal ulcer; GI, gastrointestinal; NSAID, nonsteroidal anti-inflammatory drug
